# Blind Interleaver Parameters Estimation Using Kolmogorov–Smirnov Test

**DOI:** 10.3390/s21103458

**Published:** 2021-05-15

**Authors:** Seungwoo Wee, Changryoul Choi, Jechang Jeong

**Affiliations:** Department of Electronic Engineering, Hanyang University, Seoul 04763, Korea; slike0910@hanyang.ac.kr (S.W.); denebchoi@gmail.com (C.C.)

**Keywords:** interleaver, blind interleaver parameters estimation, non-cooperative systems, linear block codes, channel coding

## Abstract

The use of error-correcting codes (ECCs) is essential for designing reliable digital communication systems. Usually, most systems correct errors under cooperative environments. If receivers do not know interleaver parameters, they must first find out them to decode. In this paper, a blind interleaver parameters estimation method is proposed using the Kolmogorov–Smirnov (K–S) test. We exploit the fact that rank distributions of square matrices of linear codes differ from those of random sequences owing to the linear dependence of linear codes. We use the K–S test to make decision whether two groups are extracted from the same distribution. The K–S test value is used as a measure to find the most different rank distribution for the blind interleaver parameters estimation. In addition to control false alarm rates, multinomial distribution is used to calculate the probability that the most different rank distribution will occur. By exploiting those, we can estimate the interleaver period with relatively low complexity. Experimental results show that the proposed algorithm outperforms previous methods regardless of the bit error rate.

## 1. Introduction

The use of error correcting codes (ECCs) is essential for designing reliable digital communication systems. However, while ECCs helps to accurately transmit and receive information for random errors, it has relatively low probabilities to to correct burst errors. To improve the error correction performance for burst type errors, an interleaving technique is used to convert burst errors occurring in the channel into random errors [1].

For reconstructing the real bitstreams, the receiver has to find the structure and parameters of the channel code. A blind interleaver parameters estimation must be performed first, following which the other parameters can be easily deduced.

A deinterleaving problem is usually considered in a non-cooperative context. Considering that receivers only know the intercepted sequences, blind interleaver parameters estimation algorithms exploit the fact that the codewords generated by ECCs have linear dependence [2,3,4,5,6].

In [2], Gaussian elimination is used to find the interleaver parameters. However, because this method does not consider channel noise, the performance of estimation deteriorates steeply as the number of errors increases. A blind interleaver parameters estimation algorithm that uses Gauss-Jordan elimination through pivoting and considers channel noises is described in [3]. This method finds almost dependent columns which have the minimum Hamming weight and uses it as a measure for predicting interleaver period.

Some algorithms use the rank distribution of random square matrices, ρ, as a criterion for blind interleaver parameters estimation [4,5,6]. In [7,8], they estimate interleaver parameters using the variation of rank ratio depending on the column length of the matrix. These algorithms are based on the fact that the rank distribution of square matrices consisting of codewords, $\rho_{C}$, is far different from ρ. Because ρ follows a specific distribution, the interleaver period of the intercepted bitstreams can be estimated by comparing this distribution with $\rho_{C}$.

In [4], when *s* represents rank deficiency factor, which is the difference in ranks between the rank of the *l* × *l* random binary square matrix, Λ, and *l*, they defined success events as the rank deficiency *s* is greater than 2, s>2. The number of success events is used as a measure to estimate the interleaver periods by modeling ρ as a Bernoulli trial that classifies events into success and failure based on the rank values.

In addition, the Kullback–Leibler divergence (KLD) was used as a measure for estimating the interleaver parameters [5]. KLD calculates the degree of rank distribution difference between random square matrices and square matrices of the intercepted stream. Further, KLD was also partially applied for false alarm control. In [9], maximum difference selection (MDS) is proposed by selecting vectors having fewer errors and they adopted KLD to control false alarm rates.

As an extension to [4], more events are considered using Multinomial distributions [6]. Because multinomial distributions can calculate the probability that each event will occur, it predicts interleaver periods by finding the event that has the lowest probability. According to the methodology of the analytical and histogram approach, zero mean ratio values are used to estimate an interleaver period in [7]. Recently, normalized non-zero-mean-ratio values are introduced as a measure for estimation of interleaver period [8]. Also, blind interleaver parameters estimation algorithms have been proposed focused on the case of a short length of received data [10,11]. In order to overcome degrading estimation performance caused by the lack of available data, they generated additional data by combining received data [10]. Then, the collected and generated data were used to construct matrices. The rank deficiency of the matrices can be utilized to estimate interleaver periods. In addition, the new approach estimating interleaver periods without generating additional data has been proposed when the length of the received data is short [11]. They focused on the case that the data collected was so short that matrices could not be sufficiently constructed to calculate rank deficiencies. To solve this problem, the matrix created once from the received data is split into multiple submatrices to calculate the rank deficiency.

In this paper, we do not consider the case of short received data and propose an improved approach that utilizes the difference between the random binomial distribution and the rank distribution of matrices composed of codewords. For a blind interleaver parameters estimation, we used the Kolmogorov–Smirnov (K–S) test. K–S statistics has been used in the automatic modulation classification (AMC) algorithm to measure the fitness whether two groups are extracted from the same distribution [12,13]. We utilized the K–S test as a metric to find the final candidate of interleaver peroids with the most different rank distribution from the rank distribution of the random signals. We utilize the K–S test as a metric to make decision whether two groups are extracted from the same distribution. The K–S test value is used to find the most different rank distribution. The K–S test does not include any logarithmic calculation and instead needs only the cumulative distribution function (CDF) of two distributions. Therefore, differences between the two distributions can be easily computed without requiring the complex logarithmic operation that is part of the KLD.

Experimental results validated that our proposed algorithm has relatively low computational complexity and outperforms existing algorithms. Furthermore, we can finer control false alarm rates compared to other algorithms by using multinomial distribution. In a noisy channel environment, simulation results demonstrate that the K–S test estimates the interleaver parameter with high estimation accuracy.

The rest of this paper is organized as follows. Related work is described in Section 2 In Section 3, the proposed algorithm is explained. The proposed algorithm is compared with conventional algorithms and analyzed based on experimental results in Section 4. Finally, Section 5 conclude the paper.

## 2. Materials and Methods

This section introduces some backgrounds to give a basic concept for understanding the proposed algorithm. We introduce the need of interleaver parameter estimation. First, the reason why a system for interleaver parameter estimation is needed in a non-cooperative context. Through the following subsections, we show the rank distribution of random square matrices, ρ, and explain conventional algorithms.

### 2.1. Interleaver Parameters Estimation for Deinterleaving Sequence

**Figure 1:**
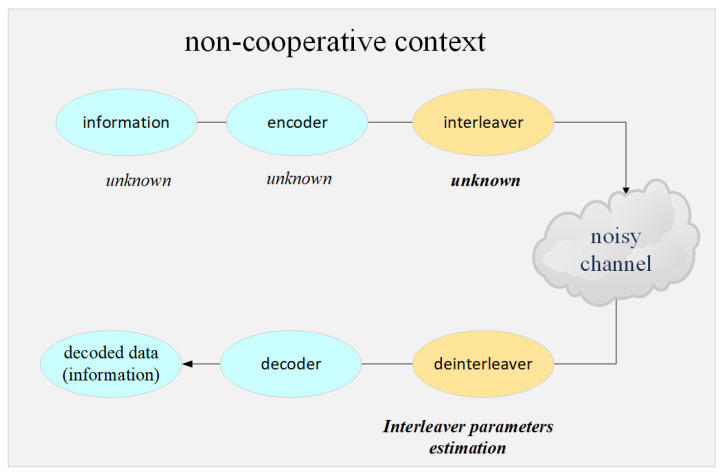
Illustration of the process of interleaver paramters estimation.

The system configuration for estimating interleaver parameter estimation in a non-cooperative context can be represented as shown in Figure 1. For ECC, encoded information is transmitted after the encoded data is interleaved. In non-cooperative context, receivers indirectly collects the transmitted signals and they do not have prior information about encoded data or parameters of interleaver. Therefore, the deinterleaving process is essential for receivers to decode the collected data.

**Figure 2:**
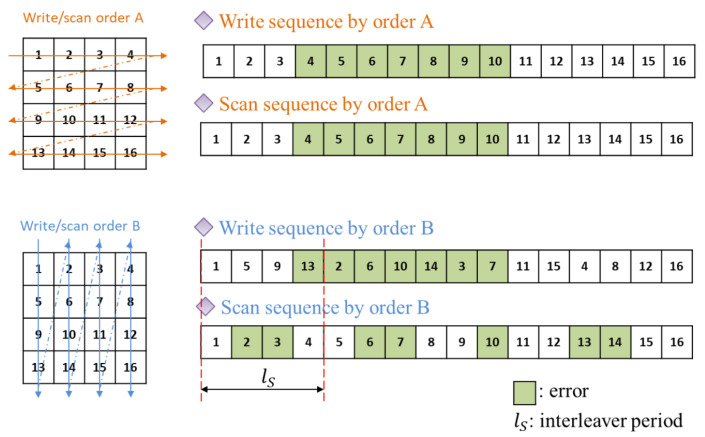
Illustration of block interleavers when the peroid of block interleaver $l_{s}=4$.

In this paper, we focus on block interleavers and introduce some existing methods to predict the interleaver duration. Block interleaving refers to arranging encoded data columns in a certain block unit and then transmitting them by changing columns and rows. Figure 2 shows the difference in results according to whether or not block interleaving is applied when a burst error occurs. Through the block interleaver, it can be seen that the 7-bit continuous burst error occurring on the channel is spread evenly on the receiver side. In general, the linear dependence of codewords have been used to estimate interleaver parameters [2,3,4,5,6,9,10,11].

### 2.2. Rank Distributions of Square Matrices and Linear Dependence of Codewords

Assume that there is l×l square matrices, Λ, whose elements follow a uniform distribution. The rank distributions of the Λ are computed as described in [14]. As *l* increases, the rank distribution quickly converges to a specific distribution.

The rank distributions can be calculated as shown in Table 1 when l→∞ [15]. $P_{s}$ is the probability that the rank of a random binary square matrix is l−s. It decreases steeply with increasing *s*(s>1). In Table 1, when s>4, $P_{s}$ is relatively very small and has no significant effect, so we consider $P_{s}\left(s=4\right)$ as $P_{s}$(s≥4).

A totally different probability distribution from that given in Table 1 is obtained when the elements of matrices are not random signals but codewords. In channel coding, an (n,k) linear block code has a generator matrix that has a full rank, where *n* is the codeword length and *k* is the length of the message. The block code generates codewords and there is linear dependence among codewords because they form a vector space [16].

Since block codes are typically specified for correcting wide-spread i.i.d. errors over the block, the performance of the receiver deteriorates when burst errors occur. To spread burst errors over the received bitstream, the encoded bits are interleaved by every certain period of time. The interleaving interval is typically a multiple of the codeword length. In other words, the interleaver period can be represented as S=βn where β∈N+.

Given the intercepted symbols, we can divide the bitstream by the length of *l* and construct Λ. If l=S, the rows of the Λ have a linear dependence because the row elements are composed of codewords. Therefore, the rank distributions of the Λ are far different from that shown in Table 1.

Based on the linear dependence of the codewords, the ranks of the Λ can be used as a criterion for estimating interleaver periods. This can be used to distinguish the two distributions from matrices consisting of random binary signals and codewords [4,5,6].

### 2.3. Bernoulli Trial Based Method

By using the rank deficiency factor *s* as an interleaver period estimator, the strict failure of interleaver period prediction can be determined when (s<2) is observed base on Table 1. This simplifies the problem of interleaver parameters estimation to counting the success events that follows the binomial distribution [4].

The constructed Λ from intercepted symbols can be considered as a random square matrix, a failure case, when the predicted interleaver period is not equal to the real interleaver period. On the contrary, when the estimated period matches the real one, the number of success cases increases owing to the high probability of rank deficiency.

Finally, the predicted interleaver size *l* is declared only when the maximum number of success events exceeds a certain threshold for false alarm control.

### 2.4. Multinomial Distributions Based Method

In [6], a rank distribution is calculated by counting the rank of the constructed Λ from the received bitstream *N* times. Using the distribution, the probability of occurrence of the rank distribution can be modeled by multinomial distribution as follows:

(1) $$P_{l\times l}=\frac{N!}{x_{0}!x_{1}!x_{2}!x_{3}!}P_{0}^{x_{0}}P_{1}^{x_{1}}P_{2}^{x_{2}}P_{3}^{x_{3}}$$

where $P_{s}\left(s\leq 3\right)$ follows Table 1, $x_{s}$ represents the counted number that the rank of the constructed Λ equals to l−s, and *N* is the total number of counting ranks, $N=x_{0}+x_{1}+x_{2}+x_{3}$. Because values of the considered *s* range from 0 to 3, note that $P_{3}$ equals $1-(P_{0}+P_{1}+P_{2})$. $P_{l\times l}$ represents the distribution similarity between the length of two matrices consisting of random and received signals, respectively. It is used to decide whether the received signals consist of random signals or codewords.

When the distribution is the same as that shown in Table 1, $P_{l\times l}$ equals 1. This means that the distribution is from Λ. If the distribution is far different from that shown in Table 1, $P_{l\times l}$ decreases to 0 and we can consider that the elements of matrices are codewords.

### 2.5. Kullback–Leibler Divergence Based Blind Interleaver Parameters Estimation

When the constructed Λ is synchronized to real interleaver period, $\rho_{C}$ follows a different distribution from that given in Table 1 owing to the linear dependence among codewords. As a measure of probability matching, KLD was used to find the most different distribution from the value of random matrices for blind interleaver parameters estimation [5]. The KLD value is given by

(2) $$\sum |P\left(i\right)\log\frac{P\left(i\right)}{Q(i)}|$$

where P(i) is the probability distribution calculated from the captured matrices and Q(i) is that of random square matrices.

Equation (2) converges to 0 when the predicted interleaver period does not equal the actual one. If the greatest value of KLD is higher than a preset threshold, *l* is declared to be the interleaver period.

### 2.6. Maximum Difference Selection

In [9], they defined $D_{MDS}$ to calculate similarity of between two rank distributions from random and received signals. $D_{MDS}$ can be denoted as follow:

(3) $$D_{MDS}=\sum_{i}\left|P\left(X=x_{i}\right)-P\left(Y=y_{i}\right)\right|$$

where *P* is probability mass function (PMF), *X* and *Y* are rank deficiency of random matrices and observed rank deficiency from received signals, respectively. After trying to select vectors having fewer errors, false alarm rates control process is performed by using KLD.

## 3. Proposed Algorithm

In this section, we explain the proposed blind interleaver parameters estimation algorithm. The proposed algorithm adopts the K–S test to measure the difference between the two distributions and multinomial distribution is used for controlling the false alarm rates. After introducing the K–S test briefly, the proposed algorithm is described in detail.

The K–S test measures the fitness whether data are extracted from the same distribution [13]. In this scenario, we use this test as a measure to find the most different rank distribution. The degree of difference between the two distributions as calculated by this test is given by

(4) $$D=\underset{x}{sup}\left|P\left(x\right)-Q\left(x\right)\right|$$

where *P* and *Q* are CDFs and *sup* is the supremum that denotes to the least upper bound of a set. This metric measures the maximum difference between the two cumulative distributions.

### 3.1. Kolmogorov–Smirnov Test

**Figure 3:**
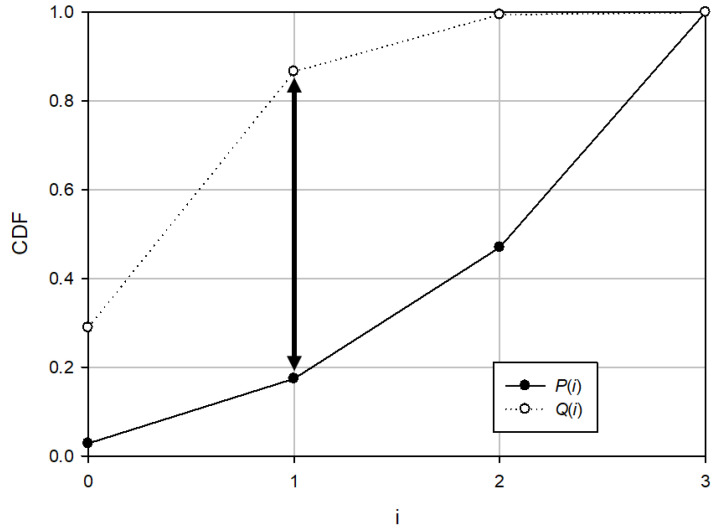
Illustration of the K–S statistic. The black arrow is the two-sample K–S statistic.

Figure 3 shows an example of the K–S statistics. The larger the K–S value, the greater is the difference between the two distributions. This motivated us to consider the K–S test as a measure for estimating interleaver parameters in our proposed algorithm. Compared to KLD, the K–S test has a relatively lower complexity. Because it does not require a logarithmic operation and needs only the CDFs of the two distributions, it can calculate the degree of difference between two distributions with low complexity.

### 3.2. Proposed Algorithm

Motivated by the foregoing observations, this subsection describes how to exploit rank distributions to estimate interleaver periods in detail. First, to exploit rank distributions for interleaver period estimation, the rank distribution must be obtained.

**Figure 4:**
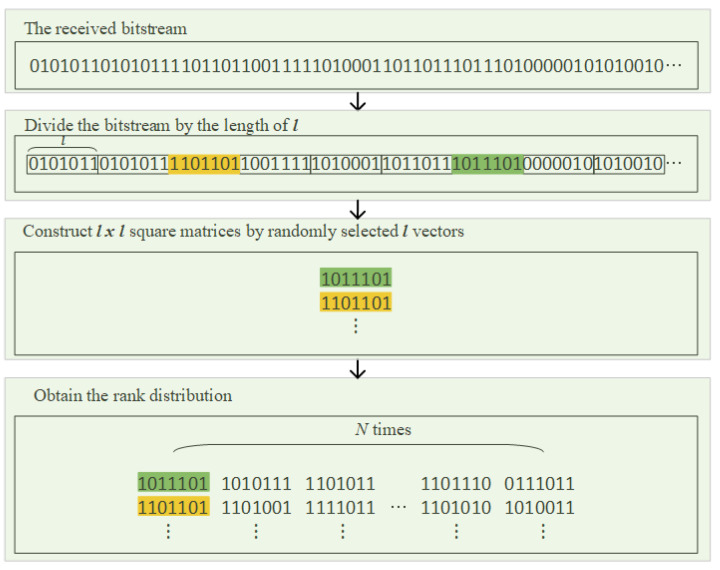
Processes used to obtain the rank distribution from the received bitstream.

We describe the process of getting rank distribution as shown in Figure 4. Given the received bitstream, *l* vectors of length *l* are randomly selected to construct Λ and the rank distribution is calculated by counting the rank of the matrices *N* times.

Sufficiently large *N* shall be considered to guarantee reliable rank distributions. For example, if *N* is set to 10,000, we must receive a captured sequence of length at least 280,000 when the interleaver period is 28. To resolve this excessive requirement, we exploited the linearity of linear codes; i.e., the sum of two codewords is also a codeword.

Then, we use the K–S test as a measure of the probability matching problem. In other words, we find *l* which has the most different distribution from that of random square matrices. Equation (5) represents how we can predict interleaver period exploiting K–S test.

(5) $$l_{S}=\underset{l}{arg max}\;D_{l}=\underset{l}{arg max}\;\underset{x_{l}}{sup}\left|P_{ran}\left(x_{l}\right)-Q_{con}\left(x_{l}\right)\right|$$

where $P_{ran}$ represents the CDF of the rank distribution from the random square matrices and $Q_{con}$ represents the CDF of the rank distribution from constructed square matrices, respectively.

If *l* is not equal to the interleaver period, *S*, there is no linear dependence in the rows of the constructed matrix. Therefore, the distribution may follow that shown in Table 1 with high probability. This results in the K–S test value, $D_{l}$, converging to near zero.

On the contrary, if *l* equals *S*, the rows of Λ consist of codewords. The distribution is totally different from that given in Table 1 owing to the linear dependence of the codewords. Therefore, $D_{l}$ can reach the maximum value and *l* can be predicted as the interleaver period.

Finally, multinomial distribution is used for false alarm control. It ensures that the probability of the rank distribution at that time is lower than a threshold, which is sufficient to judge that it does not happen by chance. The proposed algorithm is summarized in Algorithm 1.
**Algorithm 1:** Proposed Algorithm**Input**: received sequence **r**, translate parameter *d* = 0, predicted interleaver period *l* = 7**Output**: estimated interleaver period $l_{S}$1:Translate the received sequence by the length *d*.2:Divide the translated sequence by the length *l*.3:Randomly select *l* vectors and construct an *l* ×*l* square matrix Λ.4:Calculate the rank of the matrix.5:Repeat the third and the fourth steps *N* times.6:Construct rank distribution $R_{l}^{i}$7:**if**d<l−1**then** Go to the first step with increment *d* as *d*+1.8:**if**l<S+1**then** Set *d* to 0.9:Go to the first step with increment *l* as *l*+1.10:Compute (5) and declare $l_{S}$ to be an interleaver period.

## 4. Results

In this section, we present experimental results and compare the performance of the proposed algorithm to that of previous algorithms. To generate bitstreams, a (7, 4) binary Hamming code and two BCH codes, (15, 5), (15, 7), and (15, 11) were used [1]. The generated bit length was 50,000 and a random block interleaver was used.

Instead of the signal-to-noise ratio(SNR), we compare the performance of each algorithm based on the probability of errors occurring in the channel. We assumed that the interleaved bitstream passed through a binary symmetric channel with different bit error rates (BER), and even considered the possibility that the data collected by the receiver would have been translated. The number of trials for constructing rank distributions was 500 for each searching process.

For each BER, we tested the process for calculating the probability of estimating interleaver parameters 1000 times. Given the interleaver period, *S*, the search range of the predicted interleaver period, *l*, was set from 7 to S+1. The search range of the randomly chosen translation value, *d*, was set from 0 to l−1.

We compared the performance of the proposed algorithm with that of algorithms based on Bernoulli trials [4], KLD [5], Multinomial [6], and MDS [9]. In the case of Bernoulli [4], the interleaver period was predicted when the number of success events is maximum. In KLD [5], the interleaver period is predicted when (2) is maximum. Likewise, Multinomial [6] predicts interleaver periods by finding the minimum value of (1), and MDS finds the predicted length *l* with the maximum value of (3).

To show objective performance comparison, we denote detection probability as below:

(6) $$P_{D}=\frac{C_{N}}{E_{N}}$$

where $E_{N}$ is the total number of times the interleaver period is estimated, and $C_{N}$ is the number of times among $E_{N}$ predicted correctly.

**Figure 5:**
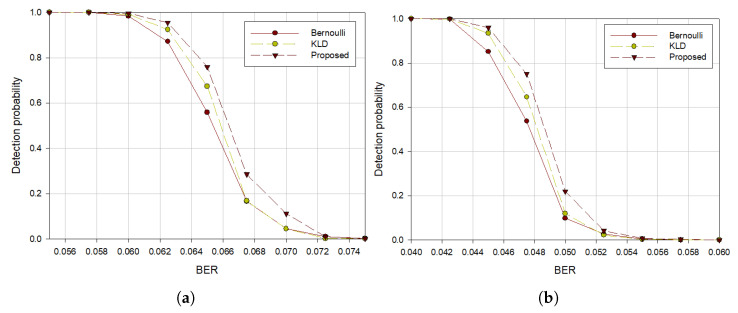
Detection probability with (**a**) BCH(15,5) code, (**b**) BCH(15,7) code when *S* = 30.

**Figure 6:**
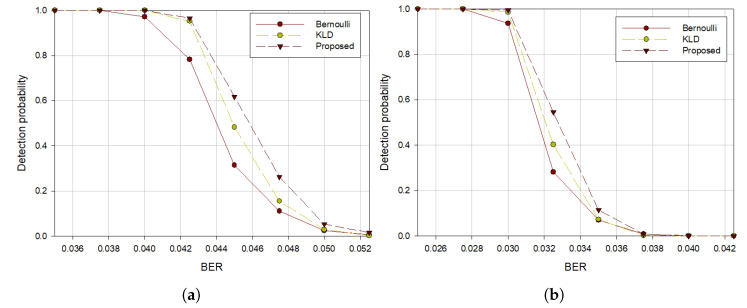
Detection probability with Hamming (7, 4) code when (**a**) *S* = 28, (**b**) *S* = 42.

**Figure 7:**
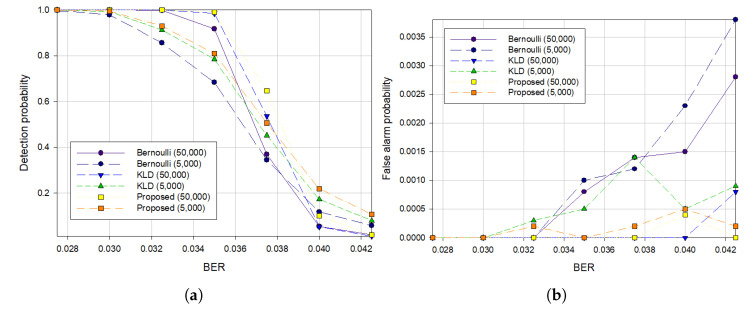
Results with Hamming (7, 4) code when S=35 (**a**) the detection probability, (**b**) the false alarm probability according to the length of bitstreams.

Figure 5, Figure 6 and Figure 7 show the experimental results in terms of code rates, interleaver periods, and the lengths of the intercepted bitstream, respectively. Since the value of (5) varies depending on the linearity of the codewords in the received data, so the higher the probability that the linearity of the codeword is not maintained, the faster the performance deteriorates according to the increase of BER.

To compare performances based on the code rates, two BCH codes were used with 50,000 intercepted symbols. As shown in Figure 5, we can see that the proposed algorithm had the highest detection probability of blind estimating interleaver parameters regardless of the code rate change. It can be seen that when the code rate is high, performance degradation begins at the lower BER than when the code rate is low. Further, Figure 5a,b show that the higher the code rates, the lower the detection probability for the same BER.

In terms of interleaver periods, we compared the performances of the proposed algorithm with the previous algorithms. Two interleaver periods, 28 and 42, were used and bitstreams of length 50,000 were generated using Hamming (7, 4) code.

Figure 6 shows that the performance of our algorithm is better than that of other algorithms in both interleaver periods. From Figure 6a,b, we observe that the performance deteriorated when the interleaver periods increased because the probability that the constructed matrices for rank calculation contain errors increases.

When comparing the performances based on the lengths of the intercepted bitstreams, we ran the test 10,000 times for each BER to achieve more accurate results. Figure 7 visualizes the detection and false alarm probability when the lengths of the bitstreams are 50,000 and 5000. When the BER is lower than 0.04, we can see that the longer the length of the intercepted bitstreams, the higher is the performance for each method.

However, when the BER is higher than 0.04, we observed that the performance with shorter intercepted symbols was the better in terms of detection probability. Further, the proposed algorithm had the lowest false alarm rates as shown in Figure 7b. Figure 7 also shows that the proposed algorithm outperformed the previous algorithms for each bitstream length.

**Figure 8:**
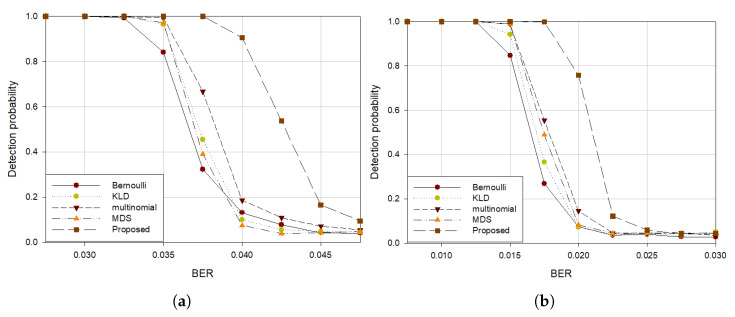
Detection probability (**a**) BCH(15, 7) code with S=35, (**b**) BCH(15, 11) code with S=35.

In addition, for a fair performance comparison with Multinomial [6] and MDS [9], additional experiments were conducted with the number of attempts to construct the rank distribution at 1000. BCH (15, 7) and BCH (15, 11) was used to show superiority of the proposed algorithm even at high code rates. Figure 8 shows that our method improved even more with increasing number of trials compared to other methods. As the code rate increases, the difference in performance from other algorithms has decreased, but it can be seen that it still outperforms the other algorithms in terms of detection probability.

Lastly, we calculated the run time for complexity comparison. Table 2 represents the time it takes to estimate the interleaver period once when S=30 with BCH (15, 11) code. The false alarm control process was omitted, and the average time was obtained by measuring the time taken to estimate the interleaver period 100 times for each algorithm.

Except for MDS [9] and Multinomial [6], Bernoulli [4], KLD [5], and proposed method have the similar execution speed as represented in Table 2. It can be seen that Bernoulli, KLD, and the proposed algorithm have a high proportion of the computational complexity of Gauss elimination process in exploiting the rank distributions. Since Multinomial [6] calculates the probability that each event will occur and finds the lowest probable event, it takes more times to estimate the interleaver periods. We can see that MDS has a relatively high computational complexity because MDS adopted the method of using vectors with a high probability of low errors before comparing two distributions.

## 5. Discussion and Conclusions

In this paper, we proposed a blind interleaver parameters estimation algorithm based on a combination of the K–S test and multinomial distribution in noisy channels. To compare the performance from various perspectives, we compared the false alarm rates according to the length of the available bitstream, the detection probability according to code rates, and execution time. The performance of the proposed estimation algorithm was superior to that of Bernoulli, KLD, Multinomial, and MDS.

We exploited the fact that rank distributions of linear codes differ significantly from those of random sequences owing to the linear dependence of linear codes. To estimate interleaver periods using that property, we adopted the K–S test as a measure of the probability matching problem between the two rank distributions of the random binary signal and the received signal. After finding the final candidate of the interleaver period, we adopted Multinomial [6] to control false alarm rates. The value of (1) is lower than a threshold, it is enough to judge that it does not happen by chance.

In terms of probability matching metric, KLD includes logarithmic operations and considers all values between two PDFs, but the our method does not involve logarithmic calculations and finds the largest difference between the two CDFs. Despite the absence of logarithmic operations, our approach took 0.14 s longer than KLD, but with shorter execution times than Multinomial and MDS.

Through the experimental results, the proposed algorithm is verified by comparing false alarm rates, the estimated performance, and execution time. In terms of false alarm rates, the probability of false alarm appears as low as KLD. We can see that our algorithm outperforms Bernoulli, KLD, Multinomial, and MDS in terms of detection probability. Also, we calculate execution time for Bernoulli, KLD, Multinomial, MDS, and our method. Although our method was the third fastest among the compared algorithm, we can see that the computational complexity of the proposed method was about 48% lower than that of MDS. Experimental results verified that our method outperformed the previous algorithms with a relatively low computational complexity.

## Figures and Tables

**Table 1:** Rank distribution of the random binary square matrices.

*s*	$P_{s}$
0	0.288788
1	0.577576
2	0.128350
3	0.005238
4	0.000048

**Table 2:** Comparison of the average run times when S=30 with BCH (15, 11) code.

Method	*t* (s)
Bernoulli [4]	11.5610
KLD [5]	11.4486
Multinomial [6]	14.3958
MDS [9]	22.3636
Proposed	11.5862

## Abbreviations & Symbols

The following abbreviations and symbols are used in this manuscript:

ECC	Error-Correcting code
K–S	Kolmogorov–Smirnov
KLD	Kullback-Leibler Divergence
MDS	Maximum Difference Selection
PMF	Probability Mass Function
CDF	Cumulative Distribution Function
AMC	Automatic Modulation Classification
BER	Bit Error Rate
ρ	Rank distribution of random square matrices
*s*	Rank deficiency factor
*l*	the row(column) length of square matrices
Λ	Rank of square matrices
$P_{s}$	Probability that the rank of a random binary square matrix is l−s
*S*	Interleaver period
